# Supplementary material for: The neural dynamics of hierarchical Bayesian causal inference in multisensory perception
Source: Nat Commun. 2019 Apr 23;10:1907. doi: 10.1038/s41467-019-09664-2 (PMC6478901; doi:10.1038/s41467-019-09664-2)
Supplement: Supplementary file 2 — Reporting Summary [file 41467_2019_9664_MOESM2_ESM.pdf]

nature research

Corresponding author(s): Tim Rohe

Reporting Summary

Nature Research wishes to improve the reproducibility of the work that we publish. This form provides structure for consistency and transparency in reporting. For further information on Nature Research policies, see Authors & Referees and the Editorial Policy Checklist.

Statistical parameters

When statistical analyses are reported, confirm that the following items are present in the relevant location (e.g. figure legend, table legend, main text, or Methods section).

n/a	Confirmed	
☐	☒	The exact sample size (*n*) for each experimental group/condition, given as a discrete number and unit of measurement
☐	☒	An indication of whether measurements were taken from distinct samples or whether the same sample was measured repeatedly
☐	☒	The statistical test(s) used AND whether they are one- or two-sided<br>*Only common tests should be described solely by name; describe more complex techniques in the Methods section.*
☒	☐	A description of all covariates tested
☐	☒	A description of any assumptions or corrections, such as tests of normality and adjustment for multiple comparisons
☐	☒	A full description of the statistics including central tendency (e.g. means) or other basic estimates (e.g. regression coefficient) AND variation (e.g. standard deviation) or associated estimates of uncertainty (e.g. confidence intervals)
☐	☒	For null hypothesis testing, the test statistic (e.g. *F*, *t*, *r*) with confidence intervals, effect sizes, degrees of freedom and *P* value noted<br>*Give P values as exact values whenever suitable.*
☒	☐	For Bayesian analysis, information on the choice of priors and Markov chain Monte Carlo settings
☐	☒	For hierarchical and complex designs, identification of the appropriate level for tests and full reporting of outcomes
☐	☒	Estimates of effect sizes (e.g. Cohen's *d*, Pearson's *r*), indicating how they were calculated
☐	☒	Clearly defined error bars<br>*State explicitly what error bars represent (e.g. SD, SE, CI)*

Our web collection on statistics for biologists may be useful.

Software and code

Policy information about availability of computer code

Data collection	Audiovisual stimuli were presented and behavioral responses were collected with Psychtoolbox 3.09 (www.psychtoolbox.org) running under MATLAB R2016a (MathWorks).<br>EEG Data was recoded with BrainVision recorder V.1.20.0802.
Data analysis	Behavioral data was anaylyzed with Matlab 2015b using custom-built code. Fitting of the Bayesian causal inference was done with custom-build Matlab code (based on Koerding et al., 2007), which is available in the G-Node repository.<br>Model comparison of computational models was done with SPM12 (spm_BMS function).<br>EEG data was preprocessed with Brainstorm 3.4. Decoding analyses were done using libSVM 3.20 (SVR decoding) and Pattern Component Modelling toolbox (RSA).<br>For analysis of parameters of the Bayesian causal inference model as a function of pre-stimulus oscillations, custom-built Matlab 2015b code was used.

For manuscripts utilizing custom algorithms or software that are central to the research but not yet described in published literature, software must be made available to editors/reviewers upon request. We strongly encourage code deposition in a community repository (e.g. GitHub). See the Nature Research guidelines for submitting code & software for further information.

Data

Policy information about availability of data

All manuscripts must include a data availability statement. This statement should provide the following information, where applicable:

- Accession codes, unique identifiers, or web links for publicly available datasets
- A list of figures that have associated raw data
- A description of any restrictions on data availability

The raw behavioral and EEG datasets generated and analyzed in the current study are available in a G-Node repository, [https://doid.gin.g-node.org/ec6518f9df39caa49d67679425224497/]. The source data underlying Figures 1-7, Tables 1-2 and Supplementary Figures 1-8 and Table 1 are provided as a Source Data file in the same G-Node repository.

Field-specific reporting

Please select the best fit for your research. If you are not sure, read the appropriate sections before making your selection.

☐ Life sciences ☒ Behavioural & social sciences ☐ Ecological, evolutionary & environmental sciences

For a reference copy of the document with all sections, see nature.com/authors/policies/ReportingSummary-flat.pdf

Behavioural & social sciences study design

All studies must disclose on these points even when the disclosure is negative.

Study description	The study is an experimental quantitative study on healthy human participants, collecting behavioral and EEG data.
Research sample	The sample consists of 24 healty human participants of which 23 were included (10 female; mean age 36.0 years, range 25-61 years). The sample was recruited among employes of the Clinic for Psychiatry and Psychotherapy, University of Tuebingen, Germany, using an email announcment. Thus, the sample was choosen to accomodate a broader age range and educational background (as compared to studies using mostly students). Yet, the sample is not representative of the whole population.
Sampling strategy	The sampling procedure was to include participants meeting the inclusion/exclusion criteria by order of response to recruitment emails, until the desired sample size was reached. A simple size calculation (using GPower) determined sufficient power using a sample size of 20 (for clear behavioral effects of the experimental paradigm). Because noisy EEG data might lead exclusion of participants, we decided to sample 24 participants.
Data collection	Psychtoolbox 3.09 (www.psychtoolbox.org) running under MATLAB R2016a (MathWorks) presented audiovisual stimuli, recorded behavioral responses and sent trigger pulses to the EEG recording system. Auditory stimuli were presented at ≈ 70 dB SPL via two loudspeakers (Logitech Z130) positioned on each side of the monitor. Visual stimuli were presented on an LCD screen with a 60 Hz refresh rate (EIZO FlexScan S2202W). EEG signals were recorded from 64 active electrodes positioned in an extended 10-20 montage using electrode caps (actiCap, Brain Products, Gilching, Germany) and two 32 channel DC amplifiers (BrainAmp, Brain Products). Electrodes were referenced to FCz using AFz as ground during recording. Signals were digitized at 1000 Hz with a high-pass filter of 0.1 Hz. Electrode impedances were kept below 25 kOhm. The researcher (TR) was the only person present during data collection. He was not blinded to the study hypothesis, but he was blinded w.r.t. to the experimental condition which participants were presented with because he was outside the EEG chamber during the experiment.
Timing	Experimental data collection started on 9th of May, 2016, and stopped on 20th of January, 2017.
Data exclusions	One participant completed only 6 experimental runs and did not attend the interview session and was thus excluded.
Non-participation	No participants dropped out/declined participation.
Randomization	Participants were not allocated in different groups.

Reporting for specific materials, systems and methods

Materials & experimental systems

n/a	Involved in the study	
☒	☐	Unique biological materials
☒	☐	Antibodies
☒	☐	Eukaryotic cell lines
☒	☐	Palaeontology
☒	☐	Animals and other organisms
☐	☒	Human research participants

Methods

n/a	Involved in the study	
☒	☐	ChIP-seq
☒	☐	Flow cytometry
☒	☐	MRI-based neuroimaging

Human research participants

Policy information about studies involving human research participants

Population characteristics	See above.
Recruitment	The sample was recruited among employes of the Clinic for Psychiatry and Psychotherapy, University of Tuebingen, Germany, using an email announcement.<br>Thus, the sample was choosen to accomodate a broader age range and educational background (as compared to many behavioral studies using mostly students). However, self-selection (e.g. only very motivated employees wanted to participate) very unlikely influences our results because the investigated effects of audiovisual integration were all within-subject effects. Differences due age, sex education, motivation etc cancel out or, if anything, lead to a larger across-participant variation, leading to more conservative tests of random-effects' significance). Yet, the sample is not representative of the whole population.
